# The effects of six-day SSRI administration on diurnal cortisol secretion in healthy volunteers

**DOI:** 10.1007/s00213-018-5050-1

**Published:** 2018-10-03

**Authors:** Amy Ronaldson, Livia A. Carvalho, Karen Kostich, Antonio Ivan Lazzarino, Livia Urbanova, Andrew Steptoe

**Affiliations:** 1 Research Department of Behavioural Science and Health, University College London, 1-19 Torrington Place, London, WC1E 6BT UK; 2 Department of Clinical Pharmacology, William Harvey Research Institute, Queen Mary University of London, John Vane Science Centre, Charterhouse Square, London, EC1M 6BQ UK

**Keywords:** Cortisol, HPA axis, Depression, SSRIs, Antidepressants, Escitalopram

## Abstract

**Rationale:**

Hyperactivity of the hypothalamic-pituitary-adrenal (HPA) axis has been widely reported in depression, and evidence suggests that selective serotonin reuptake inhibitors (SSRIs) might exert their therapeutic effects through altering cortisol secretion.

**Objective:**

This study assessed the effects of SSRI administration on diurnal cortisol secretion in healthy volunteers.

**Methods:**

Sixty-four healthy men and women were randomised to receive either 10 mg escitalopram or placebo for six days in a double-blind fashion. On day six of medication, saliva samples were obtained at home for measurement of diurnal cortisol parameters (cortisol slope, cortisol awakening response, total daily cortisol output).

**Results:**

Women receiving escitalopram had significantly steeper cortisol slopes across the day compared with those receiving placebo (*F*(1, 36) = 7.54, *p* = 0.009). This alteration in cortisol slope was driven by increases in waking cortisol levels (*F*(1, 35) = 9.21, *p* = 0.005). Escitalopram did not have any significant effect on the cortisol awakening response or the total daily cortisol output.

**Conclusions:**

Flattened cortisol slopes have been seen in depression. The results of this study suggest that escitalopram might exert its therapeutic effect in women in part through correction of a flattened diurnal cortisol rhythm.

## Introduction

Depression is one of the most common stress-related disorders. A deficit in serotonergic activity is part of the neurobiology of depression. Hyperactivity of the hypothalamic-pituitary-adrenal (HPA) axis has also been widely reported in major depression (Otte et al. 2016). There is evidence that the abnormalities of the serotonergic system and the HPA axis are linked and this interaction may be an important mechanism involved in the development of depression (Porter et al. 2004).

Cortisol is the endpoint of the HPA axis and is the major circulating glucocorticoid in humans. Studies examining the effects of selective serotonin reuptake inhibitors (SSRIs) on cortisol levels have produced varied results. In healthy and depressed participants, short- and longer-term SSRI administration has brought about both increases (Ljung et al. 2001; Sagud et al. 2002) and decreases in basal cortisol levels (Jazayeri et al. 2010; Ahmed et al. 2011; Hernandez et al. 2013; Dziurkowska et al. 2013; Park et al. 2015), and some studies have reported null effects (Mück-Seler et al. 2002; Deuschle et al. 2003; Kauffman et al. 2005). These findings suggest interaction between SSRIs and the HPA axis but allow us to infer very little about the direction of the effect.

These mixed findings likely relate to issues of cortisol measurement. A recent meta-analysis examining changes in cortisol secretion as a predictor of anti-depressant response has shown that results largely depend on the methodological quality of the study, with the timing and method of cortisol measurement being of great importance (Fischer et al. 2017). Cortisol secretion has a marked diurnal pattern. Cortisol is at high levels on waking, followed by a rise that reaches a peak approximately 30 min after waking. This is referred to as the cortisol awakening response (CAR). There is then a subsequent decline across the day, with cortisol reaching its nadir at around midnight (Adam and Kumari 2009). However, to date, studies have largely relied on single measurements of cortisol taken at varying times across the day. Failure to take the diurnal patterning of cortisol secretion into account makes it difficult to make inferences about the effects of SSRIs on HPA axis function. Sampling cortisol several times across the day allows for the measurement of the diurnal cortisol profile and a more in-depth investigation of the involvement of the HPA axis.

Dysregulation of the HPA axis can be due to a reduction in the amplitude of the diurnal pattern (i.e., a flatter slope across the day), a blunted or exaggerated CAR, or changes in total daily cortisol output (area under the curve (AUC)). Dysregulation of HPA axis function has been reported in depression. Depressed patients have been found to have both blunted and heightened CARs (Dedovic and Ngiam 2015), increased AUC (Dienes et al. 2013; Marchand et al. 2014; Boggero et al. 2017), and flatter diurnal cortisol slopes (Adam et al. 2017). It is possible that SSRIs exert their therapeutic effects through ‘normalising’ dysregulation of the diurnal pattern of the HPA axis seen in depression. However, very few studies have examined this. In patients with major depressive disorder (MDD), and in first-degree relatives of depressed patients, longer-term administration of SSRIs has been found to decrease cortisol AUC and decrease waking cortisol levels (Hinkelmann et al. 2012; Knorr et al. 2012; Ruhé et al. 2015). This small body of work suggests that SSRI treatment affects the pattern of diurnal cortisol secretion in depressed patients and those at risk of depression. However, some of the studies cited above report symptom remission alongside SSRI-induced alterations in diurnal cortisol secretion (Hinkelmann et al. 2012; Ruhé et al. 2015). In addition, studying this association in depressed patients may be confounded by symptomatic features of the disease, medications, and clinical history. This means that we cannot distinguish whether observed changes in cortisol secretion are due to symptomatology or direct biological effects of serotonergic alterations on HPA axis function. To date, only one study has assessed the effects of SSRI administration on diurnal cortisol secretion in healthy volunteers. In this study, short-term administration of SSRIs brought about increases in waking cortisol levels (Harmer et al. 2003), in direct opposition to the effects seen in patients with depression. However, Harmer et al. (2003) did not report on other parameters of diurnal cortisol secretion known to be important in depression (Adam et al. 2017).

Therefore, the aim of the current study is to assess the effects of short-term SSRI administration on several aspects of diurnal HPA axis function in healthy volunteers. We hypothesise that six-day administration of the SSRI escitalopram will bring about alterations in the CAR, the cortisol AUC, and the cortisol slope. However, we are unable to hypothesise about the direction of these alterations as this study is the first to explore the effects of SSRIs on these cortisol parameters in non-depressed people. Harmer et al. (2003) observed increases in waking cortisol after six-day administration of citalopram in healthy volunteers. Therefore, we hypothesise that escitalopram will bring about the same increase in our participants. Sex is known to be an important confounder when it comes to examining stress-related disease (Bale and Epperson 2015), and women show greater HPA axis reactivity to stress compared to men (Goel et al. 2014). Depression is more prevalent in women (Otte et al. 2016), and women have been found to be more responsive to SSRIs than men (Khan et al. 2005). Therefore, in this study, we will also examine how sex influences the effects of SSRIs on diurnal HPA axis function.

## Materials and methods

### Participants and design

The data used in this analysis were collected as part of the Stress Pathways Study. The Stress Pathways Study was a randomised, double-blind, placebo-controlled trial designed to assess the effects of seven-day administration of pharmacological probes on the stress response in healthy volunteers. All data were collected with the written informed consent of the participants. Ethical approval was obtained from the UCL Research Ethics Committee.

Participants were 70 healthy volunteers who were recruited from UCL campus. Participants were randomised to receive either 10 mg escitalopram (SSRI) or placebo every morning after breakfast for seven days. The seven-day study period was chosen, as escitalopram is known to exert therapeutic effects in patients with depression by the end of one week of treatment (Montgomery et al. 2001; Taylor et al. 2006). Saliva samples were provided at home for diurnal cortisol measurement on day six of placebo/escitalopram administration. Participants had to be generally healthy, aged 18–65 years and not taking any medications regularly (excluding the contraceptive pill). Specific exclusion criteria included any chronic haematological, inflammatory, pulmonary, liver, renal, gastrointestinal, heart, cerebrovascular, and psychiatric disease; any history of thromboembolism; and any current infection. Participants who suffered from asthma, who had known allergies to the study medications, previous gastrointestinal bleedings, or who were pregnant or breastfeeding were excluded. Only patients with blood pressure in the normal range were included (90/60 to 140/90 mmHg).

We carried out analyses on 64 participants who successfully provided saliva samples on day six (32 escitalopram, 32 placebo). Of the six participants excluded from the analysis, three failed to return the saliva samples (all placebo), and three dropped out due to side effects (all escitalopram).

### Study protocol

Study participants came to a brief session where they had their body composition measured and completed a questionnaire containing demographic and psychosocial measures. Participants then received the study medication and were instructed to take one capsule every morning after breakfast for the following seven days. Participants were advised not to take any other medications or herbal remedies while taking part in the study and to avoid alcohol and vigorous physical activity. Participants were also provided with a saliva sampling kit to be used at home in order to analyse diurnal cortisol secretion. The following morning participants began taking the medication. On day six of medication, saliva sampling for the measurement of diurnal cortisol secretion took place. Participants were recruited in a manner which ensured that saliva sampling always took place on a weekday.

### Diurnal salivary cortisol

The saliva kit included seven pre-labelled ‘salivette’ collection tubes (Sarstedt, Leicester, UK) and a cortisol diary. The cortisol diary contained instructions on how and when to give samples. These diaries were also used to record information on factors likely to introduce variation in cortisol samples such as mood, exercise, and daily stressors. Participants provided seven saliva samples over the course of a weekday: on waking, 30 min after waking (30+), 10 a.m., noon, 4 p.m., 8 p.m., and bedtime. Participants stored their sample in the refrigerator before returning them to the laboratory at UCL. Cortisol levels were assessed from saliva using a time-resolved immunoassay with fluorescence detection at the University of Dresden. Inter- and intra-assay variability was below 4%.

Following analysis, three different indices of diurnal HPA axis function were calculated for each participant: CAR, cortisol AUC, and cortisol slope across the day. The CAR was calculated by subtracting the waking from the + 30-min values. When calculating the CAR, we omitted individuals who reported a delay of > 15 min between waking and taking the ‘waking’ sample (Dockray et al. 2008). We computed the cortisol AUC with respect to ground (Pruessner et al. 2003). The cortisol slope was calculated in nanomoles per litre per minute (nmol/L/min) by regressing cortisol on sample collection time, with + 30 min excluded; higher values indicate a steeper decrease in cortisol over the day. Waking and evening (the average of 8 p.m. and bedtime) values were also calculated. As there were missing cortisol samples for some participants, the sample size for each analysis differed (Table 2).

### Stress-related psychological factors

Depressive symptoms, anxiety, and positive affect were measured at baseline and the day after diurnal salivary cortisol collection (day seven). Depressive symptoms were measured using the Beck Depression Inventory (BDI)-II (Beck et al. 1988). The Cronbach’s alpha for the BDI-II in this sample was 0.87 at baseline. Anxiety was measured using the anxiety subscale of the Hospital Anxiety and Depression Scale (HADS) (Zigmond and Snaith 1983). The Cronbach’s alpha for the HADS anxiety subscale at baseline was 0.83. Positive affect was measured using the positive subscale of the Positive and Negative Affect Scale (PANAS) (Watson et al. 1988). The Cronbach’s alpha for the positive affect scale was 0.83 at baseline.

### Demographic factors

Age, sex, ethnicity, BMI, smoking status, and level of parental education were measured in all participants. Hormonal contraceptive use was measured in women. As the majority of participants were students, parental education was used as an indicator of socioeconomic status (SES). Smoking status was measured as a binary variable (current smoker/non-smoker). BMI was calculated using the standard formula (kg/m^2^).

### Statistical analyses

Normality tests revealed that all cortisol parameters were normally distributed. Two-way ANOVAs and chi-square tests were used to compare the medication groups on all demographic characteristics. Where relevant, sex was included as a between-person factor alongside medication. Changes in stress-related psychological factors were assessed using two-way ANCOVAs, with medication and sex as between-person factors, and baseline values being included as covariates. Differences between the two medication groups on all diurnal cortisol parameters were analysed using two-way ANCOVAs, with medication and sex being included as between-person factors. We examined the main effects of medication as well as the interactive effect of sex. Where there were significant interaction effects of sex on diurnal cortisol parameters, we repeated the analyses in men and women separately. The significance level was set to *p* < 0.05 for all analyses, with precise *p* values reported for all test results. All statistical analyses were performed using SPSS version 22.0 (SPSS Inc., Chicago, IL, USA).

## Results

### Participants

Table 1 summarises the characteristics of the participants. The sample had an age range of 18–33 years, were almost two-thirds women (62.5%), and were mostly normal weight (79.7% BMI < 25). Over half the sample were white (53.1%), and the majority of participants had a high SES based upon parental education (84.4%).

**Table 1:** Demographic characteristics of the sample (*n* = 64)

	Escitalopram (*n* = 32)	Placebo (*n* = 32)	Escitalopram vs. placebo	
Characteristic	Mean ± SD or *N* (%)	Mean ± SD or *N* (%)	Group *p* value	Group × sex *p* value
Age (years)	22.06 ± 3.89	22.22 ± 3.00	0.692	0.021*
Female	17(53.1)	23(71.9)	0.121	–
BMI (kg/m^2^)	23.26 ± 4.17	23.20 ± 4.01	0.757	0.267
Smoker	9(28.1)	4(12.5)	0.120	–
Ethnicity (White)	17(53.1)	17(53.1)	0.955	–
SES (*n* = 64)			0.853	–
Low	3(9.4)	3(9.4)	–	–
Medium	1(3.1)	2(6.3)	–	–
High	27(84.4)	27(84.4)	–	–
Hormonal contraception	7(41.2)	5(21.7)	0.304	–
Depressive symptoms baseline	5.91 ± 6.59	6.41 ± 5.07	0.685	0.463
Depressive symptoms 7 days	5.67 ± 5.19	6.13 ± 6.88	0.709	0.241
Anxiety symptoms baseline	4.75 ± 2.81	5.28 ± 4.03	0.739	0.945
Anxiety symptoms 7 days	3.90 ± 2.87	5.61 ± 4.32	0.064	0.119
Positive affect baseline	35.25 ± 5.74	34.97 ± 4.81	0.548	0.128
Positive affect 7 days	32.73 ± 7.19	33.97 ± 6.01	0.385	0.230

*statistically significant (*p* < 0.05)

Scores on the BDI-II at baseline ranged from 0 to 31 indicating the presence of depression in some participants. One participant (escitalopram) had a BDI-II score greater than 19 indicating the presence of probable clinical depression. Scores on the HADS anxiety subscale at baseline ranged from 0 to 15 indicating the presence of probable anxiety in some participants. Five participants had scores of 11 or greater indicating anxiety (one escitalopram; four placebo). Sensitivity analyses were carried out with these participants removed (*n* = 5). Exclusion of these participants did not affect the results of the study.

The escitalopram group did not differ significantly from the placebo group in terms of sex, BMI, smoking status, ethnicity, or SES (see Table 2 for all *p* values). There were also no significant differences between groups in baseline depression scores, anxiety scores, or positive affect scores. Amongst female participants, there was no significant difference between experimental conditions in terms of hormonal contraception use. There was a significant interaction between medication group and sex with respect to age (*F*(1, 60) = 5.60, *p* = 0.021), with differences in age in men but not women. Men in the escitalopram group were younger (*M* = 20.06 years, SD = 0.63 years) than men receiving placebo (*M* = 22.44 years, SD = 0.81 years).

**Table 2:** Mean diurnal cortisol parameter values

	Escitalopram	Placebo	Escitalopram vs. placebo	
Diurnal cortisol parameters	Mean ± SD	Mean ± SD	Group *p* value	Group × sex *p* value
Waking cortisol (nmol/L) (*n* = 61)	25.16 ± 12.83	18.67 ± 13.34	0.071	0.104
Bedtime cortisol (nmol/L) (*n* = 62)	5.99 ± 9.68	6.31 ± 8.76	0.836	0.092
Average evening cortisol (nmol/L) (*n* = 62)	6.41 ± 5.87	6.58 ± 5.29	0.904	0.085
Cortisol AUC (nmol/L) (*n* = 59)	209.57 ± 82.49	188.31 ± 71.82	0.225	0.676
CAR (nmol/L) (*n* = 52)	7.63 ± 12.26	13.07 ± 14.85	0.440	0.221
Cortisol slope (nmol/L/min) (*n* = 62)	0.0219 ± 0.0239	0.0153 ± 0.0224	0.378	0.023*

*statistically significant (*p* < 0.05)

### Stress-related psychological factors

We investigated the effects of the study medications on depression, anxiety, and positive affect on the seventh day of administration. This was in order to clarify that any differences in diurnal cortisol parameters between drug groups on the sixth study day were not caused by changes in stress- or mood-related factors. The escitalopram group did not differ from the placebo group in depression scores, anxiety scores, or positive affect (see Table 1). There were also no main or interactive effects of sex on any of these factors.

### Diurnal cortisol parameters

There was no main effect of medication and no main or interactive effect of sex on cortisol AUC (all *p* values > 0.05). There was also no main effect of medication or interactive effect of sex on the CAR (all *p* values > 0.05). However, there was a main effect of sex on the CAR (*F*(1, 48) = 4.62, *p* = 0.037). Women had more pronounced CARs than men, regardless of medication group (Women: *M* = 13.19 nmol/L, SE = 2.39 nmol/L; Men: *M* = 5.03 nmol/L, SE = 2.95 nmol/L).

**Fig. 1:**
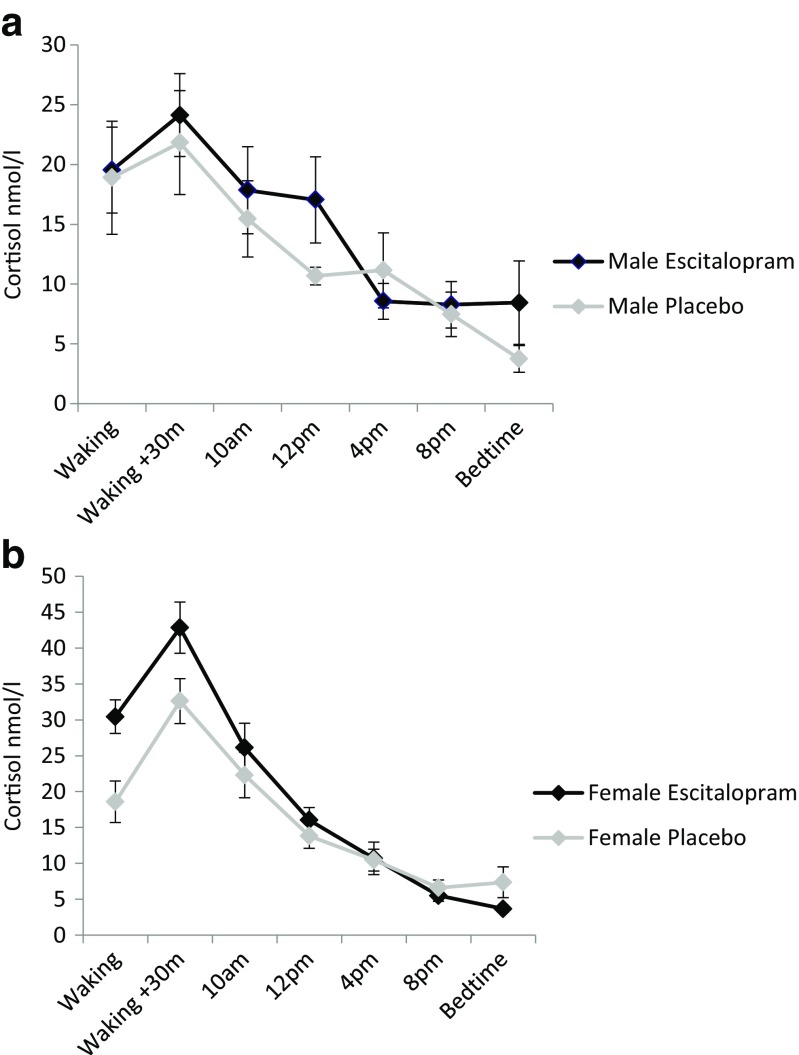
Escitalopram versus placebo. Mean salivary cortisol values across the day in **(a)** men and **(b)** women. Saliva samples were taken on waking, waking + 30 min, 10 a.m., noon, 4 p.m., 8 p.m., and at bedtime in healthy volunteers who received six days treatment with escitalopram (black line), or placebo (grey line). Error bars represent SEM

There was no main effect of medication (*p* = 0.38) or sex (*p* = 0.12) on cortisol slope. However, the ANOVA revealed a significant medication group by sex interaction effect on cortisol slope (*F*(1, 58) = 5.49, *p* = 0.023). Analysing male and female participants separately revealed no effect of medication on cortisol slope in men (*p* = 0.40). However, there was an effect of medication in women (*F*(1, 36) = 7.54, *p* = 0.009). Women taking escitalopram had steeper cortisol slopes (*M* = 0.033 nmol/L/min, SD = 0.017 nmol/L/min) compared with women receiving placebo (*M* = 0.014 nmol/L/min, SD = 0.023 nmol/L/min) (Fig. 1).

Alterations in cortisol slope can be driven by levels of cortisol at waking and/or in the evening. Therefore, we examined the effect of escitalopram on waking and evening cortisol levels in female participants. There was a significant main effect of medication on cortisol waking values (*F*(1, 35) = 9.21, *p* = 0.005), with levels being higher in female participants taking escitalopram (*M* = 30.44 nmol/L, SD = 9.39 nmol/L) than placebo (*M* = 18.57 nmol/L, SD = 13.31 nmol/L). There was no main effect of medication on cortisol evening values (*p* = 0.13). What these findings suggest is that the alterations in cortisol slope seen in the female escitalopram group were being driven by increases in waking cortisol levels.

Because men receiving escitalopram were somewhat younger on average than men receiving placebo, sensitivity analyses were carried out with age included as a covariate in the male-only analyses. ANCOVA revealed no significant main effects of drug on any of the cortisol parameters (all *p* values > 0.05).

## Discussion

The aim of this study was to assess the effects of six-day administration of escitalopram on several different indices of diurnal HPA axis function in healthy volunteers. This was the first study to examine the effects of SSRIs on cortisol slope, CAR, and AUC in healthy volunteers. This permitted the measurement of the direct effects of SSRIs on diurnal cortisol secretion independent of symptom remission or changes in mood. We hypothesised that escitalopram would lead to changes in the CAR, the cortisol AUC, and the cortisol slope, and, more specifically, that escitalopram would bring about an increase in waking cortisol levels. We also postulated that sex would influence the effects of escitalopram on cortisol. The results of this study provide some support for these hypotheses. Compared with placebo, women taking escitalopram had significantly steeper cortisol slopes across the day. This difference was independent of any differences in stress- or mood-related factors, suggesting that the observed results were due to direct biological effects of escitalopram on HPA axis function. In line with our hypothesis, this alteration in cortisol slope seen in women taking escitalopram was driven by an increase in waking levels of cortisol. Six-day administration of escitalopram did not have a significant effect on the cortisol AUC or the CAR.

It is difficult to compare our results with previous work directly, because *almost all* studies have assessed effects of SSRIs on HPA axis function in clinical populations. Cortisol AUC and the CAR are known to be altered in depression (Marchand et al. 2014; Dedovic and Ngiam 2015). Our study involved healthy volunteers which may explain why we did not observe any significant effects on overall daily cortisol output or on the CAR. However, we did find that escitalopram brought about a steepening of the cortisol slope in healthy women and that this was likely driven by increases in waking cortisol levels. This finding is supported by the work of Harmer et al. (2003) who found that six-day administration of citalopram (20 mg/day) brought about significant increases in waking cortisol in healthy volunteers. Interestingly, in depressed patients, SSRIs have been found to *lower* waking cortisol levels (Knorr et al. 2012; Ruhé et al. 2015). Since waking cortisol levels may be increased in depression (Bhagwagar et al. 2005), the direction of the effect of SSRIs on waking cortisol could be related to mental health status.

Mechanistically, there are a number of ways in which escitalopram could have altered HPA axis function in women in the current study. The serotonergic system has been found to exert substantial effects on HPA axis function (Porter et al. 2004). 5-HT receptor agonists are known to induce cortisol secretion in both human (Pitchot et al. 2004) and murine studies (Mikkelsen et al. 2004). Therefore, SSRI-induced increases in serotonin might bring about changes in cortisol secretion via the serotonergic receptors. In fact, immunohistochemical studies have shown that 5-HT receptors are present on the paraventricular nucleus of the hypothalamus which is responsible for the release of corticotropin-releasing hormone (CRH)—the initial effector of the HPA axis (Lanfumey et al. 2008). Melatonin—a hormone involved in regulating sleeping and waking cycles—has been shown to affect 5-HT receptor-mediated activation of the HPA axis (Raghavendra and Kulkarni 2000). SSRIs increase melatonin levels in depressed patients (Carvalho et al. 2009). Therefore, SSRI-induced changes in melatonin levels might explain the alterations in waking cortisol levels in the current study.

Escitalopram may have also exerted direct effects on HPA axis function. A growing body of research suggests that SSRIs may affect the HPA axis via modulation of the corticosteroid receptors. Four days treatment with citalopram has been shown to increase both glucocorticoid and mineralocorticoid receptor sensitivity in healthy humans (Pariante et al. 2004, 2012). Flatter cortisol slopes have been associated with reduced GR sensitivity (Jarcho et al. 2013). It is possible that the steeper cortisol slope seen in women taking escitalopram in the current study is a result of increased sensitivity of the corticosteroid receptors.

Steeper slopes were only observed in women taking escitalopram. There are a number of reasons why this might be. Firstly, there are known sex differences in HPA axis function (Bale and Epperson 2015). Women have been shown to have increased diurnal cortisol secretion (Carpenter et al. 2015), and higher oestrogen levels have also been associated with higher morning cortisol peaks (Wolfram et al. 2011). This may be why we observed higher CARs in women in the current study, independent of the effects of the study medications. Women have been shown previously to have higher CARs compared with men (Kunz-Ebrecht et al. 2004). Male steroidal sex hormones also appear to play a role in cortisol secretion. For example, testosterone is known to decrease corticosterone in rats (Panagiotakopoulos and Neigh 2014).

Secondly, the sex difference observed in the current study may be related to 5-HT1A receptor expression. Stimulation of the 5-HT1A receptor increases cortisol secretion (Pitchot et al. 2004). It may be that female HPA axis function is more responsive to increased levels of serotonin due to enhanced receptor stimulation. According to Goel and colleagues, oestrogen potentiates 5-HT1A receptor stimulation of the HPA axis, whereas testosterone decreases it (Goel et al. 2014). This may explain the increases in waking cortisol seen in the women receiving escitalopram. In further support of this, research has shown that the level of 5-HT1A receptor mRNA in the pituitary gland is almost seven times higher in women (Goel and Bale 2010).

Finally, women with depression are thought to have more favourable therapeutic responses to SSRIs. A review of 15 RCTs revealed that female depressed patients on the whole are more responsive to SSRI treatment than male patients in terms of symptom remission (Khan et al. 2005). There is also evidence for a role of oestrogen in the sex differences seen in therapeutic responses to SSRIs (Damoiseaux et al. 2014).

In terms of therapeutic implications, as mentioned previously, flatter cortisol slopes have been observed in depression (Sjögren et al. 2006; Jarcho et al. 2013; Doane et al. 2013). Although no changes in stress or mood factors were observed, the steepening of the cortisol slope observed in women taking SSRIs in the current study may be one of the mechanisms through which these drugs exert their therapeutic effects. HPA axis changes might precede mood effects. This area warrants further investigation, and future studies should take sex differences into consideration.

A strength of this study is that it was a randomised placebo-controlled double-blind trial. The study had a retention rate of 91.4% with 64 participants providing usable data on some parameters of diurnal cortisol secretion. However, it is possible that this study was underpowered to detect certain effects. There were more women than men in the current study, meaning that we may have lacked sufficient statistical power to detect drug effects in men. We included women in the study who were taking hormonal contraception which is known to affect cortisol secretion (Kirschbaum et al. 1999). This may have impacted results. However, there was no difference in contraception use between the two experimental conditions. Additionally, our sample was largely composed of healthy university students from high socioeconomic backgrounds. Therefore, the results may not be readily generalizable to other groups, or to clinical groups with depression. Cortisol was measured over a single day, meaning that the diurnal secretion may have been affected by situational factors rather than long-term factors. However, we measured cortisol over the course of a weekday which may help counteract the effects of single-day sampling as most people have established weekday routines. Nevertheless, this measurement issue should be borne in mind while interpreting results.

In conclusion, the results of this study indicate that six-day treatment with the SSRI escitalopram brings about a steepening of cortisol slopes in healthy women, via increases in waking cortisol levels. Flattened cortisol rhythms have been seen in depression. This finding suggests that SSRIs may exert their therapeutic effects in women via correction of a flattened diurnal cortisol rhythm.
